# Investigation of the presence of specific neural antibodies in dogs with epilepsy or dyskinesia using murine and human assays

**DOI:** 10.1111/jvim.16744

**Published:** 2023-05-26

**Authors:** Lea Hemmeter, Christian G. Bien, Corinna I. Bien, Andrea Tipold, Jasmin Neßler, Andrea Bathen‐Nöthen, Kaspar Matiasek, Maik Dahlhoff, Clare Rusbridge, Carina Rotter Black, Kai Rentmeister, Holger A. Volk, Andrea Fischer

**Affiliations:** ^1^ Section of Neurology, Centre for Clinical Veterinary Medicine LMU Munich Munich Germany; ^2^ Department of Epileptology (Krankenhaus Mara) Bielefeld University, Medical School Bielefeld Germany; ^3^ Laboratory Krone Bad Salzuflen Germany; ^4^ Department of Small Animal Medicine and Surgery University of Veterinary Medicine Hannover Foundation Hannover Germany; ^5^ Tierarztpraxis, Dr A. Bathen‐Nöthen Cologne Germany; ^6^ Section of Clinical & Comparative Neuropathology, Centre for Clinical Veterinary Medicine LMU Munich Munich Germany; ^7^ Institute of In Vivo and In Vitro Models, University of Veterinary Medicine Vienna Vienna Austria; ^8^ Fitzpatrick Referrals, Halfway Lane Surrey UK; ^9^ School of Veterinary Medicine, Faculty of Health & Medical Sciences University of Surrey Surrey UK; ^10^ Tierärztliche Praxis für Neurologie Dettelbach Germany

**Keywords:** animal model, autoimmune epilepsy, dyskinesia, epileptic seizure, idiopathic epilepsy, movement disorder, neural antibodies, neuroinflammation, sleep disorder

## Abstract

**Background:**

Autoimmune mechanisms represent a novel category for causes of seizures and epilepsies in humans, and LGI1‐antibody associated limbic encephalitis occurs in cats.

**Hypothesis/Objectives:**

To investigate the presence of neural antibodies in dogs with epilepsy or dyskinesia of unknown cause using human and murine assays modified for use in dogs.

**Animals:**

Fifty‐eight dogs with epilepsy of unknown cause or suspected dyskinesia and 57 control dogs.

**Methods:**

Serum and CSF samples were collected prospectively as part of the diagnostic work‐up. Clinical data including onset and seizure/episode type were retrieved from the medical records. Screening for neural antibodies was done with cell‐based assays transfected with human genes for typical autoimmune encephalitis antigens and tissue‐based immunofluorescence assays on mouse hippocampus slices in serum and CSF samples from affected dogs and controls. The commercial human und murine assays were modified with canine‐specific secondary antibody. Positive controls were from human samples.

**Results:**

The commercial assays used in this study did not provide unequivocal evidence for presence of neural antibodies in dogs including one dog with histopathologically proven limbic encephalitis. Low titer IgLON5 antibodies were present in serum from one dog from the epilepsy/dyskinesia group and in one dog from the control group.

**Conclusion and Clinical Importance:**

Specific neural antibodies were not detected using mouse and human target antigens in dogs with epilepsy and dyskinesia of unknown origin. These findings emphasize the need for canine‐specific assays and the importance of control groups.

**Abbreviations**
AB: antibody
ADHD: attention‐deficit/hyperactivity disorder
AMPAR: α‐amino‐3‐hydroxy‐5‐methyl‐4‐isoxazolepropionic acid receptor
APE: antibody prevalence in epilepsy
AQP4: aquaporin‐4
BBB: blood‐brain barrier
CASPR2: contactin‐associated protein‐like 2
CBA: cell‐based assay
CNS: central nervous system
CSF: cerebrospinal fluid
DCC: deleted in colorectal carcinoma
DPPX: dipeptidyl‐peptidase‐like protein 6
EEG: electroencephalography
FEPSO: feline complex partial seizures with orofacial involvement
FLAIR: fluid attenuation inversion recovery
GABAAR: γ‐aminobutyric acid receptor A
GABABR: γ‐aminobutyric acid receptor B
GAD65: anti‐glutamic acid decarboxylase 65‐kDa isoform
GlyR: glycine receptor
IF: immunofluorescence
Ig: immunoglobulin
IgG: immunoglobulin‐G
IgLON5: immunoglobulin LON5
IL‐17: interleukin 17
IVETF: International Veterinary Epilepsy Task Force
LE: limbic encephalitis
LGI1: leucine‐rich glioma‐inactivated protein 1
mGlu5: metabotropic glutamate‐5 receptor
MRI: magnetic resonance imaging
MUE: meningoencephalomyelitis of unknown etiology
NMDAR: *N*‐methyl‐d‐aspartate receptor
TBA: tissue‐based assay
VGKC: voltage‐gated potassium channel

## 1 INTRODUCTION

Epilepsy is the most common chronic central nervous system (CNS) disease in dogs affecting 0.43% to 0.82% of dogs admitted to veterinary practices.^1^, ^2^, ^3^, ^4^ A genetic cause is frequently suspected in dogs with idiopathic epilepsy, but causal epilepsy genes are only identified for rare genetic idiopathic epilepsies with an onset in young dogs corresponding to human childhood and juvenile epilepsies.^5^ The etiology of idiopathic epilepsy with onset in adult dogs remains largely unknown. There might be a complex and multifactorial etiology with an interaction of multiple genetic risk variants and environmental factors.^6^

Autoimmune mechanisms have increasing relevance in humans as a distinct etiologic category offering new therapeutic approaches.^7^, ^8^, ^9^, ^10^ Characteristic clinical features are sudden onset of psychiatric symptoms with rapid progression to epileptic seizures or movement disorders which could be associated with signal changes on magnetic resonance imaging (MRI), cerebrospinal fluid (CSF) pleocytosis, and the presence of neural antibodies in serum or CSF.^11^ Further proof for the involvement of autoimmune mechanisms comes from positive outcomes of therapeutic trials with immunosuppressives.^9^, ^12^ Therefore, the ILAE epilepsy classification includes immune epilepsy as a distinct entity,^13^ and the ILAE autoimmunity and inflammation task force recommend distinguishing between autoimmune‐associated epilepsy and acute symptomatic seizures secondary to autoimmune encephalitis.^9^

In animals, there is unequivocal evidence for autoimmune mechanisms in cats with limbic encephalitis (LE), orofacial seizures, and suggestive MRI and pathology findings associated with antibodies directed against leucine‐rich glioma inactivated protein 1 (LGI1). The disorder in cats thus parallels anti‐LGI1 LE in humans.^14^, ^15^, ^16^, ^17^ A further report refers to a cat with LE in association with deleted colorectal carcinoma (DCC, also known as netrin‐1 receptor) autoantibodies.^18^ Reports on dogs are scarce.^19^, ^20^, ^21^, ^22^ *N*‐methyl‐d‐aspartate receptor (NMDAR) antibodies are suggested in the CSF of two dogs with meningoencephalitis of unknown etiology (MUE) and one dog with multifocal signs of neurologic disease and an abscessed maxillary tooth, and in the CSF of a polar bear (*Ursus maritimus*) that drowned because of epileptic seizures and NMDAR‐antibody (AB) mediated encephalitis.^20^, ^23^ Furthermore, positive effects of prostaglandin G/H synthase 2 (PTGS2; also known as cyclooxygenase‐2, COX‐2) inhibitors in single dogs with IE and increased T helper 17 cells (Th17) support neuroinflammatory mechanisms in idiopathic epilepsy in dogs.^24^, ^25^

This study aims to screen for neural antibodies in dogs with epilepsy or suspected dyskinesia of unknown origin with commercial diagnostic murine and human assays, modified for use in dogs. We hypothesized that we would detect cross‐reacting antibodies.

## 2 MATERIALS AND METHODS

This study was performed with institutional ethical approval (#125‐16‐05‐2018). The study was designed as a prospective case‐control study. Serum and CSF samples from 120 dogs, 63 dogs with epilepsy or suspected dyskinesia and 57 controls, were collected prospectively and screened for neural antibodies with commercially available murine and human assays.

**TABLE 1:** Clinical characteristics, diagnostic work‐up, and samples for neural antibodies in pet dogs with epilepsy or dyskinesia and controls.

	No. dogs	Additional features			Diagnostic tests				Samples		Neural antibody testing		
		Acute onset^a^	Behavior changes	Immune‐mediated comorbidity^b^	MRI	CT	CSF	EEG	Serum	CSF	Serum only	CSF only	CSF/serum pairs
**Epilepsy/dyskinesia group (n = 58)**		**30**	**17**	**18**	**52**	**1**	**48**	**12**	**57**	**38**	**20**	**1**	**37**
*Epileptic seizures*	*38*	*18*	*14*	*11*	*38*	*1*	*35*	*4*	*37*	*27*	*11*	*1*	*26*
Tonic‐clonic epileptic seizures	32	15	11	10	32	—	29	2	31	22	10	1	21
Generalized	14	7	3	4	14	—	12	—	13	10	4	1	9
Focal to bilateral tonic‐clonic	9	3	3	4	9	—	8	2	9	5	4	—	5
Additional focal seizures	9	5	5	2	9	—	9	—	9	7	2	—	7
Focal epileptic seizures	6	3	3	1	6	1	6	2	6	5	1	—	5
*Suspected dyskinesia/unclassified*	*20*	*12*	*3*	*7*	*14*	—	*13*	*8*	*20*	*11*	*9*	—	*11*
Dystonia	2	1	—	—	2	—	2	1	2	2	—	—	2
Unclassified tremor/myoclonus/episodes	6	2	1	2	6	—	5	1	6	3	3	—	3
Idiopathic head tremor	12	9	2	5	6	—	6	6	12	6	6	—	6
**Control group (n = 57)**		—	—	—	**17**	**5**	**17**	—	**46**	**17**	**40**	**11**	**6**
*Neurologic disease*	*22*	—	—	—	*17*	*5*	*17*	—	*11*	*17*	*5*	*11*	*6*
Compressive myelopathy^c^	14	—	—	—	10	5	11	—	6	11	3	8	3
Lumbosacral disease	3	—	—	—	3	—	2	—	1	2	1	2	—
Fibrocartilagenous embolism	1	—	—	—	1	—	—	—	1	—	1	—	—
Meningitis/meningoencephalitis	2	—	—	—	1	—	2	—	2	2	—	—	2
Idiopathic vestibular disease	1	—	—	—	1	—	1	—	—	1	—	1	—
Unexplained lameness	1	—	—	—	1	—	1	—	1	1	—	—	1
*Non‐neurologic disease*	*25*	—	—	—	—	—	—	—	*25*	—	*25*	—	—
Cardiologic	7	—	—	—	—	—	—	—	7	—	7	—	—
Metabolic/endocrine/gastroenterologic	6	—	—	—	—	—	—	—	6	—	6	—	—
Trauma	4	—	—	—	—	—	—	—	4	—	4	—	—
Dental	4	—	—	—	—	—	—	—	4	—	4	—	—
Orthopedic	3	—	—	—	—	—	—	—	3	—	3	—	—
Dermatologic	1	—	—	—	—	—	—	—	1	—	1	—	—
*Healthy*	*10*	—	—	—	—	—	—	—	*10*	—	*10*	—	—
Preventative health care	8	—	—	—	—	—	—	—	8	—	8	—	—
Blood donor	2	—	—	—	—	—	—	—	2	—	2	—	—
**All dogs (n = 115)**		**30**	**17**	**18**	**69**	**6**	**65**	**12**	**103**	**55**	**60**	**12**	**43**

^a^ Onset was described in detail in 54 dogs. An acute onset was defined by ≥2 days with epileptic seizures or other episodes in the first 4 weeks, or cluster seizures or episodes or status epilepticus at onset.

^b^ One dog had a history of surgical removal of an abdominal teratoma and encephalomyelitis 2 years before the onset of epilepsy (not counted here). An immune‐mediated comorbidity was an exclusion criteria for control dogs.

^c^ Intervertebral disc herniation (n = 9), subarachnoid diverticula (n = 3), disc‐associated cervical spondylomyelopathy (*n = 2*).

Inclusion criteria were epilepsy or dyskinesia of unknown cause required a tier II diagnostic work‐up in line with International Veterinary Epilepsy Task Force (IVETF) tier II recommendations.^26^ Dogs were included in the epilepsy group if ≥2 generalized or focal epileptic seizures occurred ≥24 hours apart.^26^ Paroxysmal dyskinesia was considered based on characteristic clinical features of episodes and review of video footage.^27^ Episodes which were difficult to classify were also considered in this group (Table 1). We encouraged recruitment of dogs with unusual features of epilepsy/suspected dyskinesia, for example, subtle MRI signal intensity changes of undefined significance, mild unexplained CSF pleocytosis, other non‐neurologic comorbidities suggestive of inflammation/autoimmunity or behavioral changes (not postictal or adverse effects of medications).^28^ Also, few drug‐resistant dogs from breeds predisposed to epilepsy and seven dogs with idiopathic epilepsy and increased interleukin 17 (IL‐17) levels (published previously^24^) were included. Exclusion criteria were a diagnosis or suspicion of genetic epilepsy/dyskinesia because of affected family members or obvious structural brain disease, for example, neoplasia and meningoencephalitis. Information on clinical data (age, breed, sex, onset, phenotype of seizures or episodes, comorbidities) and diagnostic findings (MRI, CSF) were extracted from the medical records. Additional telephone interviews were conducted if deemed necessary to validate information. Histopathological examination was carried out on one dog.

Control dogs had other neurological diseases but no seizures or dyskinesia (22 dogs; Table 1), non‐neurological disease (25 dogs) or were healthy dogs presenting for preventative health care or as blood donors (10 dogs). Care was taken to exclude dogs with a history of epileptic seizures, neoplasia, or recent treatment with glucocorticoids as controls.

Serum and CSF were collected as part of the routine diagnostic work‐up. MRI was performed in all dogs with epilepsy or dyskinesia except six dogs with clinical signs and videos suggestive of idiopathic head tremor syndrome described previously (Table 1).^29^ Cerebrospinal fluid was collected under general anesthesia by atlanto‐occipital puncture. Routine CSF analysis (total nucleated cell counts, cytospin differential cell count, protein content, infectious disease tests as indicated) was performed. In most cases, CSF and serum (100 μL) was shipped to the immunodiagnostic laboratory, specialized for neural AB testing in human medicine, for immediate AB testing after arrival. Additional archived samples, which had been stored at −20°C or −80°C until testing, were transported frozen on dry ice.

Screening for neural antibodies was done on a tissue‐based assay (TBA) on unfixed sagittal mouse brain slices (hippocampus, brain stem, and cerebellum; Euroimmun, Lübeck, Germany), and, secondly, by a commercial cell‐based assay (CBA) biochip containing human embryonic kidney (HEK) cells transfected with human genes for different antigens of interest (Euroimmun, Lübeck, Germany): glutamic acid decarboxylase (GAD65; intraneural) and the neural surface antigens *N*‐methyl‐d‐aspartate receptor (NMDAR), γ‐aminobutyric acid receptor B (GABABR), α‐amino‐3‐hydroxy‐5‐methyl‐4‐isoxazolepropionic acid receptor‐2 (AMPAR1/2), dipeptidyl‐peptidase‐like protein 6 (DPPX), leucine‐rich glioma inactivated protein 1 (LGI1), contactin‐associated protein‐like 2 (CASPR2), glycine receptor (GlyR: now known as GLR), metabotropic glutamate‐5 receptor (mGlu5; now known as GRM5) in all dogs and IgLON5 (42 dogs with epilepsy or dyskinesia, 55 controls). GABAAR‐transfected HEK cells were not available at the time of the study. Secondary AB was a polyclonal rabbit anti‐dog immunoglobulin‐G (IgG) AB directed against canine IgG heavy and light chains (catalog no. 304‐065‐003; Jackson ImmunoResearch/Dianova) conjugated with red Alexa Fluor 594 used at a dilution of 1:100 and incubated for 30 minutes at room temperature; nuclei were counterstained with Hoechst 33342, 1:10 000. Slides were embedded with 1,4‐Diazabicyclo[2.2.2]octan. IgG positivity was confirmed with a specific anti‐canine IgG directed against the Fc fragment (Jackson ImmunoResearch, 304‐545‐008, 1:100), conjugated with the green Alexa 488 dye. Human samples with different AB reactivities served as positive control.^8^

### 2.1 Validation

For validation of negative TBA test results, four samples with immunofluorescence (IF) staining results suspicious of neuropil binding on the initial testing by TBA and 1 sample with a positive CBA but negative TBA were re‐tested together with 16 chosen negative samples from the epilepsy/dyskinesia group. TBA was performed by two examiners (masked for review) which were blinded to the initial test results and applied stringent criteria. Samples were rated in three categories: (1) negative; (2) questionable neuropil/diffuse staining; and (3) neuropil. A positive test result required a staining, specific for neuropil and comparable with positive samples from the literature^30^ and own human results with confirmed CBA positivity. Observer agreement was evaluated by Cohen kappa. For further validation of negative test results for NMDAR ABs on CBA, western blots of canine and murine brain lysates were incubated with a commercial anti‐NMDAR‐antibody (rabbit polyclonal anti‐NMDAR1; 1:1000; #AB‐0889; ABnostics, Dossenheim, Germany) with proposed specificity against canine NMDAR to identify canine NMDAR protein. For methodological details see File S1.

## 3 RESULTS

One hundred twenty dogs were recruited for the study. Five dogs, all with negative neural AB test results, were subsequently excluded because of genetic dyskinesia (1 dog, phosphoenolpyruvate carboxykinase deficiency^31^), suspicion on genetic epilepsy (2 dogs), progressive neurologic disease (1 dog), and incomplete MRI study (1 dog with epilepsy). Thus, samples from 115 dogs (58 epilepsy and dyskinesia cases, 57 controls; 43 serum‐CSF pairs, 60 sera, 12 CSF) were included in the analysis (Table 1).

### 3.1 Clinical characteristics of the epilepsy/dyskinesia group

The epilepsy/dyskinesia group consisted of 58 dogs (33 males; 25 females; median age at study inclusion 3.75 years). Median age of onset was 2.5 years for epilepsy (range 0.4‐11.7 years) and 2.0 years for suspected dyskinesia (range 0.3‐10.5 years). Onset was described in detail in 54 dogs and was fairly acute in 55.6% (30/54) with ≥2 days with epileptic seizures or other episodes, cluster seizures or episodes or status epilepticus in the first 4 weeks (Table 1); status epilepticus (2 dogs), cluster seizures (9 dogs) or cluster episodes (5 dogs) occurred already on the first day in 29.6% (16/54). Behavioral changes, which occurred independently from the seizures or episodes and unrelated to medication, were reported in 29.3% (17/58). One dog had a history of surgical removal of an abdominal teratoma and encephalomyelitis 2 years before the onset of epilepsy with tonic‐clonic seizures but the original report or specimens were unavailable for review. Other comorbidities with a presumed immune‐mediated etiology were reported in 18 dogs (31.0%; 18/58). Details on the clinical phenotype are presented in File S2.

### 3.2 MRI and CSF findings

Subtle MRI changes were evident in 53.8% of the dogs (28/52). Brain MRI revealed regional T2‐ or T2‐FLAIR hyperintensity in 13 dogs (13/52; 25%). Hyperintense signal changes appeared in the hippocampus, temporal or piriform lobes of one or both hemispheres (11 dogs), cerebellum and brainstem (1 dog), and as leukoaraiosis in one 9‐year‐old dog. Contrast enhancement was noted in five of these dogs on corresponding regions. Other findings were variable degrees of ventriculomegaly or asymmetry (14 dogs) and soft tissue attenuating/fluid material in both tympanic bullae in one dog. Analysis of CSF (48 dogs) revealed mild CSF pleocytosis in four dogs with total nucleated cell counts from 7 to 37 cells/μL (reference range 0‐5 cells/μL). There was marked CSF pleocytosis (389/μL) and increased protein content (0.82 g/L; reference range <0.3 g/L) in a fifth dog.^32^ Histopathologic examination indicated limbic encephalitis in this dog. CSF analysis was unremarkable in the others.

### 3.3 Screening for neural antibodies with TBA

Tissue‐based assays utilizing unfixed sagittal mouse brain slices which were intended as a screening test for antibodies to new neuronal antigens showed negative results for all samples from affected dogs and controls.

Validation of test results: We were unable to confirm initial suspicion on positive IF labeling in five dogs from the epilepsy/dyskinesia group (3 CSF, 2 serum samples, including the dog with a history of teratoma) when the same samples were re‐examined by blinded investigators applying stringent evaluation criteria. Thereby, re‐evaluation of 21 samples from the epilepsy/dyskinesia group (11 CSF, 10 serum) including the five dogs with questionable positive results on initial examination and 16 dogs with initially negative test results by two experienced examiners (CIB, CGB) which had been blinded to the initial test results, showed substantial agreement (Cohen kappa = 0.67) between examiners (CGB, CIB) on interpretation of IF and classification of samples (Table 2). Finally, none of the investigated materials produced a staining that could be rated as “neuropil AB.”^33^

**TABLE 2:** Interobserver agreement on evaluation of TBA: Validation showed substantial agreement (Cohen kappa = 0.67).

		CGB			
		Negative	Questionable	Neuropil	Total
CIB	Negative	11	1	0	12
	Questionable	2	7	0	9
	Neuropil	0	0	0	0
	Total	13	8	0	21
	Agreement	13	8	0	18
	By chance	7.43 857 143	4.57 142 857	0	12

### 3.4 Screening for specific neural antibodies with CBA

**FIGURE 1:**
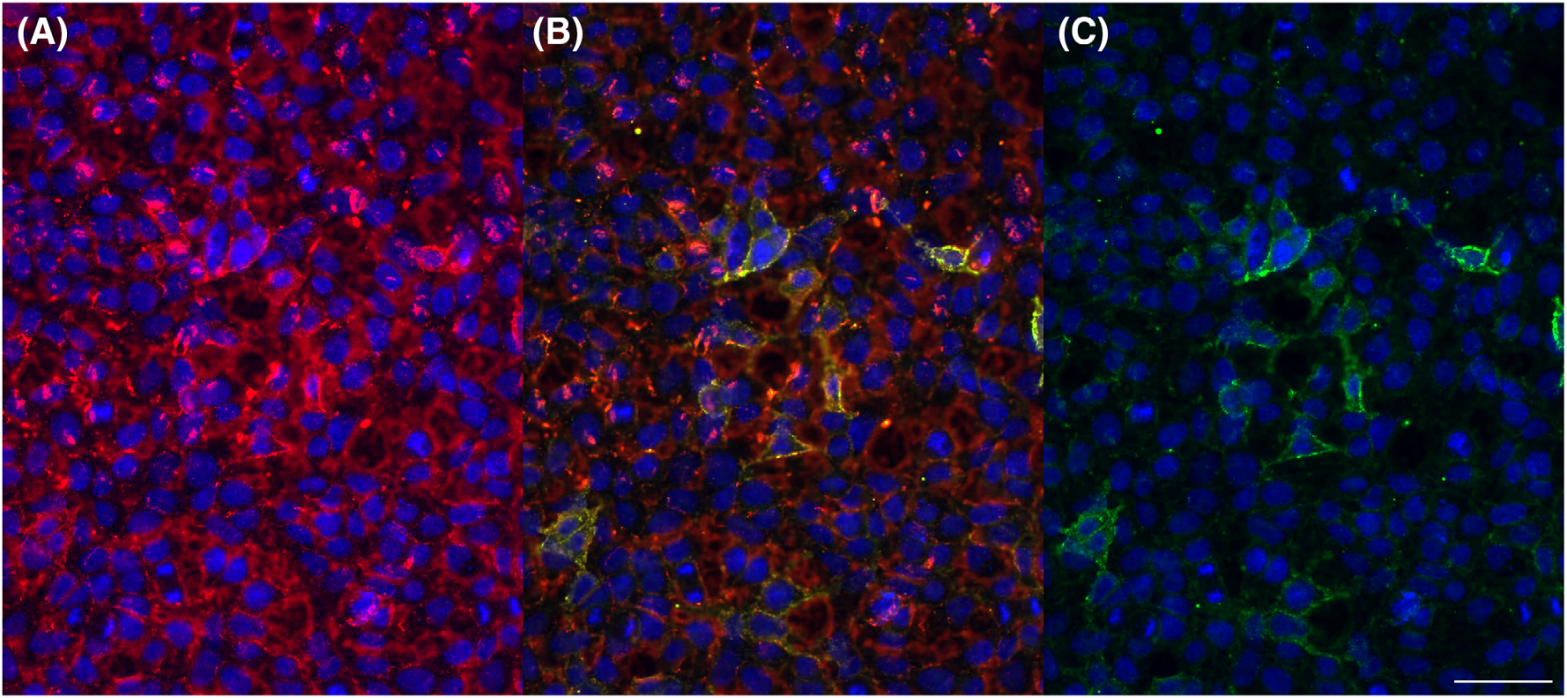
Canine anti‐IgLON5 antibodies. Immunofluorescence studies: Diluted serum (1:20) from a dog from the epilepsy/dyskinesia group (Golden Retriever, female, 3 years) is incubated with IgLON5‐transfected human embryonic kidney (HEK) cells. Binding of antibodies to cell surfaces is visualized by two different secondary anti‐canine antibodies coupled with immunofluorescence dyes and subsequently overlaid. The results demonstrate that there are canine anti‐IgLON5 IgG antibodies. Endpoint titration gave a titer of 1:40 (not shown). (A) anti‐canine IgG heavy and light chain (a sensitive secondary antibody), coupled with the red Alexa 594 dye. (B) overlay; yellow: double‐stainings. (C) anti‐canine IgG Fc fragment (the more specific secondary antibody), coupled with the green Alexa 488 dye. Bar: 25 μm.

Cell‐based assays which express human antigens provided negative results for each tested neural AB in all dogs except low serum AB titers of IgLON5 antibodies (1:40) in one dog with unclassified episodes from the epilepsy/dyskinesia group (Figure 1), but also in one dog from the control group. The case dog was a 3‐year‐old female Golden Retriever with unremarkable MRI and CSF analysis which presented for further investigation of episodes of abnormal spontaneous arousal from sleep with howling, urination, defecation followed by signs of hind limb paresis. The control dog was a 9‐year‐old German Wirehaired Pointer presenting for further evaluation of severe osteoarthritis of the elbow joint. The owner denied any signs of seizures, movement disorders, sleep‐related movements, or disturbed sleep on follow‐up phone calls. Follow‐up time was 2 years in this dog.

Both, CSF and serum were tested in the dog with a histopathological diagnosis of limbic encephalitis and the dog with a history of teratoma, and CBA provided negative results. Our attempts to further validate negative CBA test results for NMDAR antibodies on canine brain failed because the commercial NMDAR antibody did not show any specific binding in dogs while it did so in mice (File S3).

## 4 DISCUSSION

Autoimmune epilepsy is a new etiologic category of epilepsy in humans, and epilepsy of unknown cause is a frequent diagnosis in dogs.^13^, ^34^ Further investigation in dogs is warranted considering the potential implications for treatment and the high proportion of dogs with epilepsy of unknown etiology and a sudden onset in adult dogs.^4^ Therefore, in this study, we investigated neural AB profiles in serum and CSF of dogs with epilepsy and dyskinesia of unknown etiology with available commercial murine and human assays, which were adapted for the use in dogs. We aimed to include dogs with clinical features frequently associated with neural antibodies and response to immunotherapy in humans and in line with suggested diagnostic criteria in humans.^11^, ^35^

The study did not detect neural antibodies with commercial murine and human diagnostic assays in all but two dogs which showed low titers of IgLON5 ABs in serum in CBA expressing human antigen. This antibody appeared of uncertain relevance, because cell‐based assays showed IgLON5 antibodies in one dog from the epilepsy/dyskinesia group and one dog from the control group. Signs of episodic pelvic limb paresis in the IgLON5 AB‐positive dog occurred only in temporal context with sleep, that is, on awakening or associated with paradoxical arousal which is to some degree reminiscent of the frequent reports of sleep disorders in humans with IgLON5 antibodies.^36^, ^37^ We were unable to provide unequivocal classification of the episodes as dyskinesia or epileptic seizures by EEG or to perform polysomnography because of their infrequent occurrence but considering the association with involuntary urination and defecation an epileptic seizure could also have occurred. IgLON5 disease in humans is a tauopathy frequently characterized by sleep problems and signs of bulbar dysfunction as dysphagia, progressive supranuclear palsy, dysautonomia, cognitive decline, with additional signs of a movement disorder during daytime, such as distal myoclonus, chorea, limb or oromandibular dystonia, hand tremor, and facial spasm reported in up to 64%.^36^, ^37^, ^38^ We also found IgLON5 antibodies in one control dog without any signs of epileptic seizures, movement, or sleep disorder on questioning of the dog owner. This demonstrates the importance of case‐control studies to be able to judge the specificity and the pathogenic potential of neural antibodies.

The study was designed as a screening study using available human and murine assays. The study did not provide evidence for neural antibodies including NMDAR antibodies in dogs with epilepsy of unknown cause or dyskinesia with consideration of autoimmune epilepsy with human and murine assays. Thus, with the present diagnostic methods the question remains yet unresolved whether autoimmune epilepsy is a rare condition in dogs or whether the applied test methods developed for use in humans are not reliable for investigation of dogs. Considering the selection of the dogs, 1 concern is that the results could have been negative because of methodological issues, that is, that dog antibodies did not bind to human and mouse neural antigens. This hypothesis is in line with other authors' negative findings when investigating glial fibrillary acidic protein (GFAP) autoantibodies in dogs with similar methods.^21^ Furthermore, use of human immune assays for detection of antibodies to new antigenic targets in dogs is significantly limited by the lack of canine positive controls, and, therefore, we were required to use positive human samples with different AB reactivities as controls to ensure reliable staining. Another explanation might be that more recently discovered antigens like GABAAR were not available at the time of testing. Recently, we described a 1‐year‐old male intact Cavalier King Charles Spaniel with GABAAR AB, serum titer 1:320, CSF titer 1:2 and a corresponding staining pattern on mouse brain. This dog recovered upon steroid treatment from its acutely emerging and progressive signs like tonic‐clonic epileptic seizures, episodic hyperexcitability alternating with episode of stupor, and intermittent circling behavior.^22^

Even though the respective genes have amino acid sequences that are to ≥94% identical between humans and dogs,^20^ our results highlight the need for cross‐species validation of antibodies and target antigen epitopes, considering that the specificity of the AB is a critical part of IF‐based methods, and the requirement to confirm positive results from IF and diagnostic assays with other or multiple detection methods. We attempted this with western blots but surprisingly the commercial NMDAR antibody failed to recognize canine NMDAR antigen despite being marketed for dogs (Figure S3). It is a drawback that further confirmation, for example, with TBA failed in our IgLON5 AB positive dogs, and other detection methods, for example, gel electrophoresis was not pursued.^39^ In the present study, all samples were tested in a two‐step procedure, first by TBA, a screening test for unknown autoantibodies on unfixed sagittal mouse brain slices, and second by CBA, assuming that the TBA could serve as a screening test for yet unknown autoantibodies.^8^ However, the TBA has been recently questioned in humans, based on demonstration of a weak correlation between CBA and TBA with no neuropil staining in the TBA despite a clear positive CBA, which also reflects our results in the IgLON5‐positive dogs. A similar discrepancy between weakly positive CBA and negative TBA in dogs with MUE was recently described by others evaluating GFAP antibodies with TBA and CBA in dogs.^21^ Furthermore, we were unable to provide evidence for reliable neuropil staining on blinded re‐evaluation of samples with TBA. Likewise, in humans, diagnosis of autoimmune CNS disease has only been achieved in a few cases with neuropil staining in the TBA.^8^ As a consequence, the presence of yet unknown autoantibodies in dogs with epilepsy or dyskinesia remains to be elucidated with different methodology, as the TBA on its own is not an appropriate test for finding new antibodies and confirming results of CBA.

Previously, others had explored a subset of neural antibodies (NMDAR1, AMPAR1, AMPAR2, GABABR1, GABABR2, CASPR2, LGI1, and DPPX) in 32 dogs with CNS disease, including four dogs with epilepsy, with a commercially available CBA expressing human antigen and indirect immunofluorescence assay, with postulated AB specificity because of high homology of the amino acid sequences between humans and dogs.^20^ The authors reported on NMDAR1 antibodies in CSF of three dogs (9.4%). They used a less stringent approach classifying samples from two dogs with MUE with diffuse staining in CBA as positive while staining was convincing in one dog with tooth root abscesses and multifocal signs of neurologic disease which were responsive to immunotherapy but in the absence of MRI or CSF changes indicative of meningoencephalitis.^20^ Pan et al reported on high prevalences of NMDAR AB in different mammalian species, including dogs and cats, ranging from 4% in dogs <4 years of age to 30% in dogs >11 years.^40^ These authors did not consequently differentiate between Ig classes; thus comparison to our data is difficult. Differences are in part because of the fact that Pan et al used a protein‐A‐antibody detection method that did not differentiate between IgA and IgG antibodies.^40^ In contrast we used anti‐canine IgG antibody as a secondary antibody, because only IgG NMDAR ABs are shown to be of clinical relevance in humans.^41^ Interestingly, Pan et al emphasized that the immunologically relevant portions of the NMDAR were identical across species.^40^ Furthermore results might also depend on the interpretation by the authors. Misinterpretation of non‐specific cell staining as indicating antibody‐positivity has been criticized previously.^42^ We rated immunofluorescence in a stringent way. Figure 1C of the present article demonstrates the lack of background staining with a more specific anti‐IgG AB in the modified cell‐based assay.

Our affected group included dogs with epilepsy of unknown etiology or suspected dyskinesia, and we included dogs with unusual features, for example, new onset and rapid progression in adult dogs, behavioral and autoimmune comorbidities, reports on MRI signal changes in some of the dogs and inflammatory CSF changes in few dogs. Furthermore, nearly one‐third of the dog owners reported notable behavioral changes. In three dogs the onset of behavioral changes which were described by the owners as restlessness, hyperactivity, lack of concentration, and inattention was concurrent with the onset or progression of the epilepsy suggesting a shared pathophysiology between seizures and behavioral changes.^43^ In humans, sudden onset of alteration of behavior (delusions, psychosis, catatonia) and cognition combined with abnormal movements (eg, orofacial dyskinesia) are frequently seen in patients with AB against NMDAR1.^7^, ^44^, ^45^ The AB prevalence in epilepsy score (APE score) includes aside from new‐onset epileptic seizures, neuropsychiatric changes, for example, agitation, aggressiveness, emotional lability, as common symptoms of patients with positive AB‐status (up to 78.3%), and additionally rapidly progressive mental status changes within 1 to 6 weeks.^46^

At this stage, it is premature to exclude autoimmune mechanisms in dogs with epilepsy and dyskinesia of unknown origin based on negative test results for neural antibodies with assays expressing murine and human neural antigens, considering that we included one dog with epilepsy and a history of a teratoma, which is frequently associated with NMDAR1 antibodies in humans.^47^ We also included one dog with a histopathologic diagnosis of limbic encephalitis, which is frequently associated with LGI1 or CASPR2 antibodies in humans.^13^, ^30^ We were unable to identify neural AB, including LGI1 AB, in the 1 histopathologically confirmed case with LE. Low titer IgLON5 antibodies were present in 1 case and 1 control dog. Although we appreciated some parallels in clinical manifestations to IgLON5 disease in humans the finding of a low positive titer in a control dog argues against a significant association with neurologic disease in dogs. Lastly, AB status is not necessarily needed to assume immune‐mediated LE in humans and 7% of patients with clinical and MRI features of LE, CSF, and serum remain negative for neural antibodies.^48^ Furthermore, autoimmune encephalitis with epileptic seizures can also present with normal brain MRI or CSF results in humans and cats.^7^, ^16^, ^49^, ^50^

## 5 CONCLUSION

Our results highlight the need for the development of canine species‐specific assays for neural antibodies and also the need for inclusion of control groups to assess whether there is a significant association with disease.

## CONFLICT OF INTEREST DECLARATION

Andrea Fischer receives sponsoring support from Purina Deutschland GmBH (Euskirchen, Germany) for the Ludwig‐Maximilians‐Universitat epilepsy consulting unit and from Vetoquinol GmbH (Ismaning, Germany) for the Ludwig‐Maximilians‐Universitat veterinary mobility center. No other authors declare a conflict of interest.

## OFF‐LABEL ANTIMICROBIAL DECLARATION

Authors declare no off‐label use of antimicrobials.

## INSTITUTIONAL ANIMAL CARE AND USE COMMITTEE (IACUC) OR OTHER APPROVAL DECLARATION

Approved by the ethical commission of the veterinary faculty of Ludwig‐Maximilians‐Universitat Munich (No. 125‐16‐05‐2018).

## HUMAN ETHICS APPROVAL DECLARATION

Authors declare human ethics approval was not needed for this study.

## Supporting information


**File S1.** Methods western blot.

**File S2.** Details on the clinical phenotype.

**Figure S3.** Western blot of canine and murine brain lysate. Western blots demonstrating failure of the antibody to bind to NMDAR1 protein in canine brain samples while there is expression of NMDAR1 protein (100 kDa) in murine hippocampus (lane 5‐6), and murine amygdala (lane 7‐8) samples. ACTB (45 kDa) was used as reference protein.
