# Supplementary material for: Investigation of the presence of specific neural antibodies in dogs with epilepsy or dyskinesia using murine and human assays
Source: J Vet Intern Med. 2023 May 26;37(4):1409–17. doi: 10.1111/jvim.16744 (PMC10365065; doi:10.1111/jvim.16744)
Supplement: Supplementary file 1 — File S1. Methods western blot. [file JVIM-37-1409-s002.pdf]

Supporting File S1

Hemmeter et al. Investigation of the presence of specific neural antibodies in dogs with epilepsy or dyskinesia using murine and human assays.

Journal of Veterinary Internal Medicine 2023 DOI: 10.111/jvim.16744

Methods

Western blot analysis

For protein extraction, the brain tissue was homogenized in RIPA-extraction-buffer (Cell Signaling, Ipswich, USA), and the protein content was estimated by the bicinchoninic acid (BCA) protein assay. 20µg of total protein were separated by 10% SDS-PAGE and transferred to PDVF membranes (Millipore, Billerica, USA) by electroblotting. Membranes were washed in Tris-buffered saline solution with 0.1% Tween-20 (TBS-T) and blocked in 2% w/v fat-free milk powder for 1 h at room temperature. Then membranes were washed again in TBS-T and incubated in 5% w/v BSA of the appropriate primary antibody overnight at 4 °C. As primary antibodies we used rabbit polyclonal anti-NMNDAR1 (1:1000; #AB-0889; ABnostics, Dossenheim, Germany). After washing, membranes were incubated in 5% w/v fat-free milk powder with a horseradish peroxidase labelled secondary antibody (goat anti rabbit; 1:5000; #7074; Cell Signaling) for 1 h at room temperature. Immunoreactive bands were visualized by chemiluminescence with an ECL kit (Thermo Fisher Scientific) in an ECL ChemoStar Imager (Intas Science Imaging, Göttingen, Germany). After detection, membranes were stripped (Elution buffer: 2% SDS, 62.5 mM Tris/HCl, pH 6.7 and 100mM beta-mercaptoethanol for 40 min at 70 °C) and incubated with a second antibody recognizing ACTB as a control (rabbit polyclonal anti-ACTB; 1:5000; #4970; Cell Signaling, Ipswich, USA).
