# Supplementary material for: Investigation of the presence of specific neural antibodies in dogs with epilepsy or dyskinesia using murine and human assays
Source: J Vet Intern Med. 2023 May 26;37(4):1409–17. doi: 10.1111/jvim.16744 (PMC10365065; doi:10.1111/jvim.16744)
Supplement: Supplementary file 2 — File S2. Details on the clinical phenotype. [file JVIM-37-1409-s001.pdf]

Supporting file S2

Hemmeter et al. Investigation of the presence of specific neural antibodies in dogs with epilepsy or dyskinesia using murine and human assays.

Journal of Veterinary Internal Medicine 2023 DOI: 10.111/jvim.16744

Details on the clinical phenotype

Epileptic seizures (38 dogs):

Median age of onset was 2.5 years (range 0.4—11.7 years; 7 dogs ≥ six years; one dog with unknown age). Thirty-two dogs had generalized epileptic seizures (GTC). Cluster seizures or status epilepticus occurred in 25 dogs (65.8 %; 25/38). Focal signs were reported as focal onset evolving to generalized seizures (7 dogs), additional focal seizures (8 dogs, motor), or both (2 dogs). Focal onset signs were described as motor (6 dogs), motor and fly-catching behaviour (1 dog), or autonomic (2 dogs, vomiting, vomiting/vocalization). Additional unclassified episodes were episodes of staring into a void (2 dogs), head tremor (2 dogs), and orofacial twitching with whole-body tremors (1 dog). Six dogs showed only focal seizures described as episodes of hypersalivation (2 dogs), fly-catching (1 dog), impaired awareness and focal motor signs or eyelid myoclonus (3 dogs). Focal seizures were further supported by epileptiform discharges on EEG in two of these dogs.

Episodes of dyskinesia and/or unclassified episodes (20 dogs):

Median age of onset of dogs was 2 years (range 0.3—10.5 years; 3 dogs ≥ six years). Eight dogs showed episodes suspicious of dyskinesia in association with episodes of head tremor: facial, masticatory, and abdominal muscle twitches, moaning/yawning behaviour, eye movements (3 dogs); limb tremor spreading from one front limb to the ipsilateral pelvic limb (1 dog); stiffening with left-sided head turn, kyphosis,

tonic spasms or lifting of the limbs, shaking (1 dog); whole-body tremor and masticatory muscle myoclonus (1 dog); severe whole-body tremor, falling over, extension of individual limbs (1 dog); abnormal arousal from sleep with episodes of ataxia and non-ambulatory pelvic limb paresis in association with loud vocalization (howling), loss of urine and questionable responsiveness (1 dog). Additional episodes of limb tremors occurred in three dogs while standing. Episodes disappeared in association with heat (four weeks before, 2 weeks after) in one dog. In twelve dogs, episodic head tremor was the only clinical sign (4 French bulldogs, 2 Labrador retriever, 2 Doberman pinscher, 4 English bulldogs, 2 mixed breed dogs).

Behavioural changes:

Behavioural changes were reported in 29.3 % (17/58; 13 epilepsy, 4 suspected dyskinesia). Behavioural reports from the owners comprised uncontrollable hyperactivity and restlessness (8 dogs), aggression (5 dogs; described as aggressive behavior towards the owner during epileptic seizures in association with fearful behavior e. g. hiding in dark corners, growling and barking; unspecified aggression not related to epileptic seizures; new onset of aggression towards another female dog in the family; late onset of owner-directed aggression 3 years after beginning of epilepsy, and unspecified aggression), anxiety (1 dog), hyperactivity and anxiety (1 dog), hyperactivity, attention-seeking behaviour (1 dog), episodic staring (1 dog). In two of the hyperactive/restless dogs, owners complained also about signs of cognitive decline during nose-working for hunting (improved in one dog with levetiracetam).

Onset of behavioural signs was concurrent with the onset of epilepsy in two dogs, and concurrent with the progression of the epilepsy from a single generalized seizure every six months to frequent cluster seizures in one dog. In two other dogs, owners reported pronounced behavioural changes for prolonged periods i. e. two weeks after each generalized seizure.

Other comorbidities:

One dog had a history of surgical removal of an abdominal teratoma and encephalomyelitis two years prior to the onset of epilepsy but the original report or specimens were unavailable for review.

In 18 dogs (31.0 %; 18/58), other comorbidities with a presumed immune-mediated etiology were reported: food allergy (5 dogs), atopic dermatitis (2 dogs), atopy and hypothyroidism (1 dog), inflammatory bowel disease, history of other chronic gastrointestinal signs, chronic otitis externa, and dermatitis (8 dogs), hypothyroidism, flea-allergic dermatitis, otitis externa and PB-associated immune-mediated neutropenia (1 dog) and laboratory indicators of gluten hypersensitivity (1 dog).
