# Supplementary material for: Investigation of the presence of specific neural antibodies in dogs with epilepsy or dyskinesia using murine and human assays
Source: J Vet Intern Med. 2023 May 26;37(4):1409–17. doi: 10.1111/jvim.16744 (PMC10365065; doi:10.1111/jvim.16744)
Supplement: Supplementary file 3 — Figure S3. Western blot of canine and murine brain lysate. Western blots demonstrating failure of the antibody to bind to NMDAR1 protein in canine brain samples while there is expression of NMDAR1 protein (100 kDa) in murine hippocampus (lane 5‐6), and murine amygdala (lane 7‐8) samples. ACTB (45 kDa) was used as reference protein. [file JVIM-37-1409-s003.pdf]

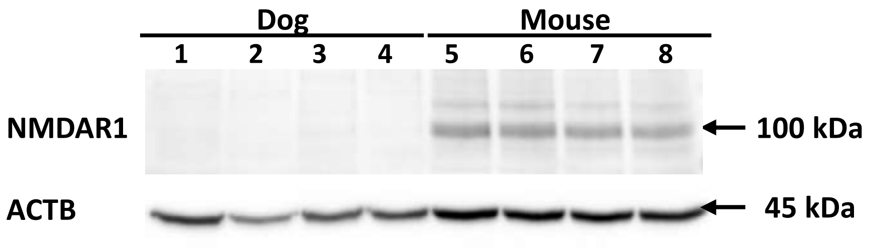

Supporting file S3. Western blot of canine and murine brain lysate. Western blots demonstrating failure of the antibody to bind to NMDAR1 protein in canine brain samples while there is expression of NMDAR1 protein (100 kDa) in murine hippocampus (lane 5-6), and murine amygdala (lane 7-8) samples. ACTB (45 kDa) was used as reference protein.

Hemmeter et al. Investigation of the presence of specific neural antibodies in dogs with epilepsy or dyskinesia using murine and human assays. Journal of Veterinary Internal Medicine 2023 DOI: 10.111/jvim.16744
